# Catatonia Psychopathology and Phenomenology in a Large Dataset

**DOI:** 10.3389/fpsyt.2022.886662

**Published:** 2022-05-23

**Authors:** Eleanor Dawkins, Leola Cruden-Smith, Ben Carter, Ali Amad, Michael S. Zandi, Glyn Lewis, Anthony S. David, Jonathan P. Rogers

**Affiliations:** ^1^South London and Maudsley NHS Foundation Trust, London, United Kingdom; ^2^Department of Biostatistics and Health Informatics, King’s College London, London, United Kingdom; ^3^Department of Neuroimaging, King’s College London, London, United Kingdom; ^4^University of Lille, Inserm, CHU Lille, U1172 – LilNCog – Lille Neuroscience & Cognition, Lille, France; ^5^UCL Queen Square Institute of Neurology, University College London, London, United Kingdom; ^6^University College London Hospitals NHS Foundation Trust, London, United Kingdom; ^7^Division of Psychiatry, University College London, London, United Kingdom; ^8^Institute of Mental Health, University College London, London, United Kingdom

**Keywords:** catatonia, psychopathology, phenomenology, subjective experience, cluster analysis, principal component, fear, anxiety

## Abstract

**Background:**

The external clinical manifestations (psychopathology) and internal subjective experience (phenomenology) of catatonia are of clinical importance but have received little attention. This study aimed to use a large dataset to describe the clinical signs of catatonia; to assess whether these signs are associated with underlying diagnosis and prognosis; and to describe the phenomenology of catatonia, particularly with reference to fear.

**Methods:**

A retrospective descriptive cross-sectional study was conducted using the electronic healthcare records of a large secondary mental health trust in London, United Kingdom. Patients with catatonia were identified in a previous study by screening records using natural language processing followed by manual validation. The presence of items of the Bush-Francis Catatonia Screening Instrument was coded by the investigators. The presence of psychomotor alternation was assessed by examining the frequency of stupor and excitement in the same episode. A cluster analysis and principal component analysis were conducted on catatonic signs. Principal components were tested for their associations with demographic and clinical variables. Where text was available on the phenomenology of catatonia, this was coded by two authors in an iterative process to develop a classification of the subjective experience of catatonia.

**Results:**

Searching healthcare records provided 1,456 validated diagnoses of catatonia across a wide range of demographic groups, diagnoses and treatment settings. The median number of catatonic signs was 3 (IQR 2–5) and the most commonly reported signs were mutism, immobility/stupor and withdrawal. Stupor was present in 925 patients, of whom 105 (11.4%) also exhibited excitement. Out of 196 patients with excitement, 105 (53.6%) also had immobility/stupor. Cluster analysis produced two clusters consisting of negative and positive clinical features. From principal component analysis, three components were derived, which may be termed parakinetic, hypokinetic and withdrawal. The parakinetic component was associated with women, neurodevelopmental disorders and longer admission duration; the hypokinetic component was associated with catatonia relapse; the withdrawal component was associated with men and mood disorders. 68 patients had phenomenological data, including 49 contemporaneous and 24 retrospective accounts. 35% of these expressed fear, but a majority (72%) gave a meaningful narrative explanation for the catatonia, which consisted of hallucinations, delusions of several different types and apparently non-psychotic rationales.

**Conclusion:**

The clinical signs of catatonia can be considered as parakinetic, hypokinetic and withdrawal components. These components are associated with diagnostic and prognostic variables. Fear appears in a large minority of patients with catatonia, but narrative explanations are varied and possibly more common.

## Introduction

In his 1874 description of catatonia, Kahlbaum designated classic objective motor and behavioural signs still used in diagnosis today, including posturing, waxy flexibility, immobility and negativism, but he also made observations on his subjects’ inner world: “the general impression conveyed by such patients is one of profound mental anguish, or an immobility induced by severe mental shock.” (1) Despite research showing that catatonia is prevalent in up to 10% of psychiatric inpatients (2), there have been few studies examining the structure of the psychopathology and fewer investigating the subjective experience. This paucity of research may be partly explained by under-recognition of catatonia (3) and the difficulties associated with many patients with catatonia presenting with mutism and negativism.

In terms of the objective psychopathology, some previous studies have endeavoured to apply principal component analysis to elucidate the structure of what is often displayed as a long list of rather disparate catatonic clinical signs (4–10). Sample sizes have varied between 106 and 2,703 and all have been in a mixed clinical population of patients with and without a diagnosis of catatonia. These analyses have reduced the structure of catatonic signs to between two and six factors; this was often based on selecting factors with an eigenvalue greater than 1, but the method of model selection was not always clear. Models with fewer factors have tended to distinguish positive and negative (or hyperkinetic and hypokinetic) signs with or without a factor for qualitatively abnormal movements (4–7). Models with more factors have been more varied, including components such as excitement or agitation, volitional disturbance, abnormal movements, inhibition, automatic movements, repetitive movements or echophenomena, grimacing and autonomic disturbance (8–10).

It is important to recognise that catatonia has been conceptualised in other ways by psychiatric schools. The Wernicke-Kleist-Leonhard school has given most attention to psychomotor function, but—rather than using an atheoretical approach based solely on symptoms and signs like that of ICD and DSM—it is based on the longitudinal course of psychiatric disorders and their family history (11). Thus there are various catatonia phenotypes, of which the best studied is periodic catatonia, which is primarily characterised by the presence of affect disturbances in both poles. A core feature of periodic catatonia is the disorganisation of psychomotor functions, which also manifests as akinesia and hyperkinesia existing in combination but in different body parts (12).

Two important questions regarding the psychopathology of catatonia remain unresolved in the literature. The first is the extent to which particular catatonic signs associate with the diagnoses underlying the catatonia. One retrospective study of 40 patients with catatonia in a general hospital found that stereotypy, mannerism, waxy flexibility and impulsivity were each more common where catatonia was due to a psychiatric disorder (such as depression or schizophrenia), compared to cases where catatonia was due to an underlying medical condition (such as autoimmune encephalitis or a space-occupying lesion) (13). Another study of 140 patients with catatonia in a specialist hospital found that patients with psychotic disorders had more posturing than those with a medical disorder, but there were no differences between those with psychotic and those with affective disorders (14). Such studies, however, are susceptible to problems of multiple comparisons when associations are tested with numerous clinical signs. An alternative approach is to use principal component analyses to summarise the clinical features in a small number of dimensions. When Krüger and colleagues examined their four-factor model, they found that patients with schizophrenia had more abnormal involuntary movements/mannerisms and disturbance of volition/catalepsy, while those with mania showed more catatonic excitement and those with depression exhibited more catatonic inhibition (8). In the six-factor model of Stuivenga and Morrens, patients with a psychotic disorder scored higher on stereotypy/mannerism, negative and excitement factors than patients with a substance use disorder or miscellaneous diagnosis; when compared to patients with mood disorders, those individuals with a psychotic disorder had higher excitement scores than those with major depressive disorder and lower excitement scores than those with bipolar affective disorder (10).

The second question regarding catatonia psychopathology concerns the phenomenon of psychomotor “alternation.” While periods of agitation in catatonia were noted at least as far back as Kraepelin (15), the description of alternation between stuporous and excited states has recently been advanced by Shorter and Fink (16). However, to our knowledge, there has been no systematic examination of the phenomenon of alternation in any large sample to define its prevalence.

In terms of the subjective phenomenology of catatonia, several studies have found an association with an intense feeling of anxiety or fear (4, 17–21), with some authors theorising the behaviours seen are akin to an evolutionary prey response to danger (21). Northoff and colleagues retrospectively asked 24 patients about their experience after recovering from an episode of catatonia and half reported uncontrollable and overwhelming emotions, in particular fear (18). In a follow-up study that compared the experience of catatonia with that of Parkinson’s disease, the researchers found that patients with catatonia were less aware of their motor deficits than patients with Parkinson’s and much more aware of emotional distress, suggesting a different underlying neural pathway leading to similarities in motor outcomes (17). The study also identified two subgroups of catatonia: emotive (high levels of anxiety and intense emotions) and non-emotive (meaningful narrative—predominating ambivalence). Those in the emotive group were more likely to respond to lorazepam treatment, which has led some researchers to suggest that the efficacy of benzodiazepines in catatonia may be mediated by their anxiolytic effect (17).

However, recent research examining catatonia in the elderly has also found that whilst anxiety is often present in catatonia, it does not explain all symptom dimensions and the types of symptoms seen may depend on the underlying cause of the catatonia (4). In this study, which used a principal component analysis, “excitement” (with loading from excitement, grimacing, echopraxia, stereotypy, mannerisms, verbigeration, grasp reflex, and combativeness), and “parakinetic” items (with loading from automatic obedience and ambitendency) were not associated with intense anxiety, whereas anxiety was positively correlated with an “inhibition” factor (with loading from immobility, mutism, staring, posturing, rigidity, withdrawal, and perseveration) and was more commonly observed in depression. Others have proposed that in addition to an anxiety response, catatonic symptoms may be a result of responses to delusional beliefs, but this hypothesis is supported by only a few case reports at present (22).

Research into the psychopathology and phenomenology of catatonia is an important area of investigation for several reasons. Firstly, understanding the underlying emotional state of patients with catatonia may help to explain why a wide range of psychiatric and general medical conditions can end in a similar psychomotor end-point. Secondly, given that the mechanisms of treatment and prediction of treatment response are not yet well-defined, it is plausible that psychopathological and phenomological factors may provide some insight into this field. Finally, since a study by Northoff et al. suggests many patients with catatonia are mute yet most recall their experiences (17), having a better understanding of the inner world of a person with catatonia may help clinicians to provide empathetic care and appropriate reassurance, enhancing the therapeutic relationship.

In this study, we aimed to:

1. Describe the psychopathology of catatonia by detailing the frequency of individual catatonic signs, performing a cluster analysis and principal component analysis of catatonic signs.
2. Assess whether the principal components of catatonia psychopathology are associated with patient demographics, underlying diagnosis, treatment setting, duration of admission and risk of subsequent catatonia relapse.
3. Describe the phenomenology of patients with catatonia by qualitative classification of patients’ descriptions of their experiences of catatonia.

## Methods

### Study Design

We conducted a retrospective descriptive cross-sectional study to examine the psychopathology and phenomenology of catatonia. We also conducted a cohort study to examine the association between various aspects of catatonia psychopathology and subsequent clinical outcomes.

### Setting

The study was conducted in a large mental health trust in South London, United Kingdom, which serves a local population of 1.3 million people (as well as national specialist services) and includes inpatient, outpatient, consultation-liaison and home treatment services. Electronic healthcare records for patients in this trust have been anonymised and entered into a searchable research database, termed the Clinical Records Interactive Search (CRIS) system (23). This system incorporates unified electronic healthcare records, which were introduced between 2005 and 2006, as well as some legacy records dating back to 1999. It now covers data for more than 500,000 individuals. The CRIS system has been approved by the Oxfordshire C Research Ethics Committee (ref: 18/SC/0372) and this study was approved by the CRIS Oversight Committee (ref: 17-102).

### Participants

We have previously identified a large cohort of 1,456 patients with catatonia from the CRIS database and have described their demographics, clinical characteristics, recreational drug use, laboratory test results and structural neuroimaging findings (24–26). In brief, this cohort was selected by sequentially conducting a search in the free text of the notes for the string “cataton*,” using a natural language processing app to remove clearly irrelevant entries followed by investigators manually screening notes. Cases were included if a diagnosis of catatonia had been made by a clinician, a date for the diagnosis was available and there was clear evidence for the presence of at least two features of the Bush-Francis Catatonia Screening Instrument (BFCSI).

### Variables

All items on the Bush-Francis Catatonia Screening Instrument (BFCSI) were scored as present or absent by one of three investigators in the original study during the course of the first clinician-identified episode of catatonia. The BFCSI and the related Bush-Francis Catatonia Rating Scale have been shown to have high validity and reliability (27–30). In our study there was substantial inter-rater reliability on the individual elements of the BFCSI, as measured by a Cohen’s kappa coefficient of 0.64 averaged across the signs (31).

Demographic variables (age, gender, and ethnicity) were taken from the structured fields of the electronic healthcare record. Age in years was defined at the onset of catatonia. Ethnicity responses were grouped according to the preferred categories of the UK Office for National Statistics (32). Diagnoses were originally coded using the International Statistical Classification of Diseases and Related Health Problems 10th Revision (ICD-10). Primary diagnoses were then grouped together as follows: organic disorders (F0, non-F-code), neurodevelopmental disorders (F7, F8, F90, F95), non-affective psychosis (F2), mood disorders (F3), neurotic disorders (F4), personality and behavioural disorders (F5, F6, F91-F94, F98) and substance use disorders (F1). Where a diagnosis had been given prior to or including the onset date of the catatonia, the latest such diagnosis was used. Where no diagnosis had been given prior to or including the onset date of the catatonia, the earliest diagnosis after the onset of catatonia was used.

For the cohort study, the outcomes were duration of admission and catatonia relapse. Duration of admission was defined for those individuals who were psychiatric inpatients as the time in days from admission to hospital discharge. Catatonia relapse was defined as the presence of a further episode of catatonia after the index case being recorded in the electronic healthcare record.

### Statistical Methods

Statistical analysis was conducted using Stata MP (RRID:SCR_012763) version 15.1 and the threshold for statistical significance was set to *p* < 0.05.

Simple descriptive statistics were used to characterise the age, gender, ethnic group, diagnosis and treatment setting of the patients as well as the frequency of individual catatonic signs. Catatonic signs were only recorded and presented for the first catatonic episode.

A hierarchical cluster analysis using Ward’s linkage was conducted for the items of the BFCSI using the *matrix dissimilarity* and *clustermat* commands. The number of clusters was decided by inspection of the cluster dendrogram.

Principal component analysis was conducted using the *pca* command. Visual inspection of the scree plot was initially used to try to ascertain the optimal number of components. Since there was no obvious elbow in the scree plot, a model with all components with an eigenvalue >1 was investigated. In this model, there were five components and we used a cut-off value for item loading of 0.30. Where an item loading for a clinical feature was >0.30 for more than one component, it was assigned to the component where it showed the highest loading. The component loadings in the five component model are shown in Supplementary Table 1. On examining this model, no clinical features were assigned to component 4; component 5 was not adequately clinically interpretable and was not consistent with the cluster analysis. A model with only two components seemed less consistent with the scree plot and disregards the very strong loading of withdrawal on the third component. We therefore decided to make a model using the first three components.

To assess associations between these principal components on the one hand, and with demographic and clinical variables on the other hand, the principal component scores were used as the outcomes of linear regression models for numerical independent variables (age), a *t*-test for binary independent variables (gender) and ANOVAs for categorical independent variables (ethnicity, diagnostic group, and treatment setting). For the cohort study, scores derived from the principal components were used as the independent variables in linear regression models with admission duration as the dependent variable and logistic regression models used catatonia relapse as the dependent variable. Results were presented unadjusted and after adjustment for age, gender, ethnicity and diagnostic group. Duration of admission was highly positively skewed, so this variable underwent a logarithmic transformation prior to linear regression and coefficients were subsequently exponentiated.

The presence of phenomenological categories was used in logistic regression models to test associations with (separately) age, gender, ethnicity, and diagnostic group.

Because the presence of two features of the BFCSI was an inclusion criterion, there were no missing psychopathology data, although we made the assumption that a feature that was not mentioned was not present. Phenomenological data were only present for a small minority of participants, whose demographic and clinical variables were similar to the overall sample. Since the purpose of the phenomenology part of the study was mainly descriptive, no adjustment to the analysis was conducted to account for missing data.

### Phenomenological Analysis

Text extracts where there was documentation of patients’ subjective experience of catatonia were noted whilst items from the BFCSI were coded. To code the phenomenological data, the authors established several *a priori* categories in which to classify text extracts, based on the literature. These were the timing of the report of subjective experience of catatonia (contemporaneous or retrospective), whether the individual was aware of or recalled the catatonia, whether they experienced distress during the catatonia, and whether they had a meaningful narrative or causal explanation for the catatonia. One author (ED) then examined all the text extracts in the light of the *a priori* categories, then in an iterative process, developed further categories as indicated by the data. A second author (LC-S) then used the categories already developed to separately code each of the text extracts, blinded to the first author’s coding. Where there was ambiguity in interpretation of a text extract, either author referred back to the complete text of the patient’s notes for context. Where there were discrepancies between the two authors, they met to agree on a consensus, involving a third author (JPR) where there was no agreement.

## Results

### Description of Sample

Table 1 provides descriptive statistics for the demographic and clinical details of the 1,456 patients (2,130 episodes) in the overall sample, as well as providing data for the subgroup of 68 patients (68 episodes) in whom phenomenological data were available.

**TABLE 1:** Description of sample.

	All patients	Subgroup with phenomenology data
Number of patients	1,456	68
Number of episodes	2,130	68
Number of patients meeting DSM-5 criteria	586	40
Age at first episode, mean (S.D.)	35.4 (16.2)	33.1 (14.0)
**Gender, *n* (%)**		
Men	803 (55.2)	40 (59)
Women	653 (44.8)	28 (41)
**Ethnicity, *n* (%)**		
White	497 (34.1)	21 (31)
Asian/Asian British	93 (6.4)	4 (6)
Black/African/Caribbean/Black British	701 (48.1)	34 (50)
Mixed/Multiple ethnic groups	49 (3.4)	3 (4)
Other ethnic groups	87 (6.0)	4 (6)
Not stated	29 (2.0)	2 (3)
**Diagnosis at first episode, *n* (%)**		
Organic disorders ^a^	54 (3.7)	0 (0)
Neurodevelopmental disorders	48 (3.3)	3 (4)
Schizophrenia and related disorders	643 (44.2)	30 (44)
Mood disorders	235 (16.1)	14 (21)
Neurotic disorders	54 (3.7)	3 (4)
Personality and behavioural disorders	25 (1.7)	3 (4)
Substance use disorders	30 (2.1)	2 (3)
Not stated	367 (25.2)	13 (19)
**Treatment setting, *n* (%)**		
Psychiatric inpatient ward	681 (46.8)	33 (49)
Community	294 (20.2)	9 (13)
General hospital	154 (10.6)	6 (9)
Crisis resolution and home treatment team	40 (2.7)	2 (3)
Health-based place of safety	22 (1.5)	2 (3)
Not stated	265 (18.2)	16 (24)

*^a^Organic disorders included dementia, delirium, organic catatonic disorder, and organic delusional disorder.*

### Frequency of Catatonic Signs

**FIGURE 1:**
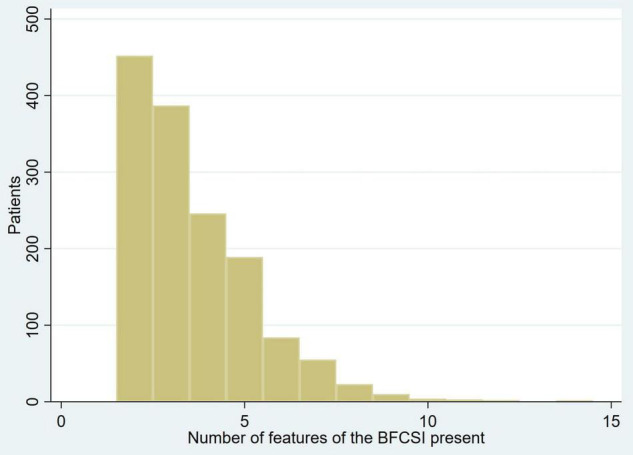
Number of catatonic features per patient (measured at first episode).

**FIGURE 2:**
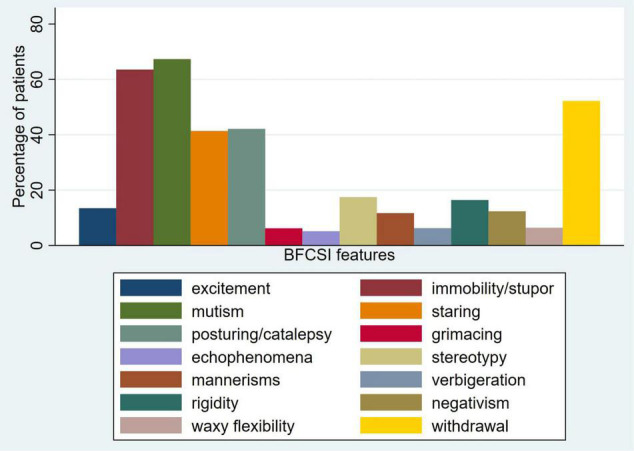
Frequency of individual items of the Bush-Francis Catatonia Screening Instrument.

The median number of features of the BFCSI was 3 (IQR 2–5, mean 3.6, SD 1.7). The number of catatonic features per patient is displayed in Figure 1. Figure 2 shows the frequency of individual catatonic features.

Since excitement and immobility/stupor may be regarded as opposite states, we examined the extent to which they co-existed in the same patients. Immobility/stupor occurred in 925 patients, of whom 105 (11.4%) also exhibited excitement; excitement was only present in 196 patients, but 105 (53.6%) of these also exhibited immobility/stupor.

### Cluster Analysis

**FIGURE 3:**
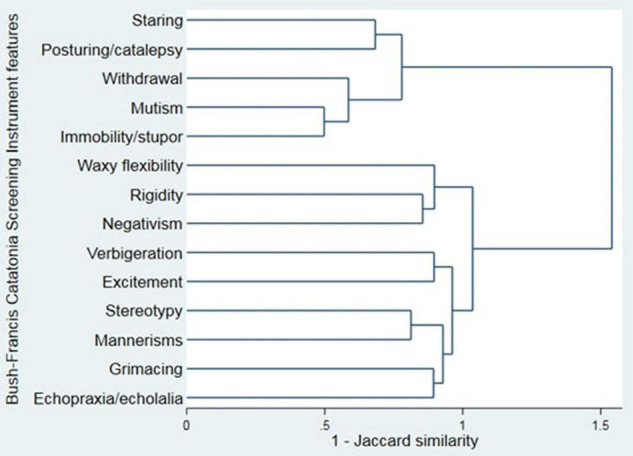
Cluster analysis dendrogram of the Bush-Francis Catatonia Screening Instrument (Ward’s linkage).

Cluster analysis was performed for the items of the BFCSI using Ward’s linkage (Figure 3). Visual inspection of the dendrogram in Figure 3 suggested a 2 component solution, as subsequent splits were much closer together. Clustering using alternative algorithms was hard to interpret, likely due to the binary nature of the data (single linkage and average linkage) or gave a similar result to Ward’s linkage (complete linkage).

If 2 clusters are used, these are a “negative” cluster (consisting of staring, posturing/catalepsy, withdrawal, mutism, and immobility/stupor) and a “positive” cluster (consisting of waxy flexibility, rigidity, negativism, verbigeration, excitement, stereotypy, mannerisms, grimacing, and echopraxia/echolalia). One thousand four hundred and forty-one (99.0%) patients had at least one feature from the negative cluster and 590 (40.5%) had at least one feature from the positive cluster.

### Principal Component Analysis

**FIGURE 4:**
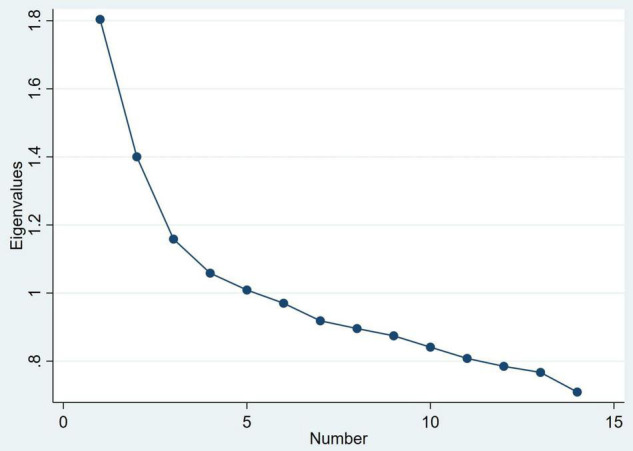
Scree plot of eigenvalues for principal components of the Bush-Francis Catatonia Screening Instrument.

A principal component analysis was used to produce a scree plot (Figure 4). As described in the Methods, a 3-component structure was chosen, which explained 31.1% of the variance. Component loadings are shown in Table 2. Posturing/catalepsy, grimacing, echopraxia/echolalia, stereotypy, and mannerisms had high loading on component 1, suggesting that this represents parakinetic features. Immobility/stupor, mutism, rigidity, negativism, and waxy flexibility had high loading on component 2, suggesting that this represents hypokinetic features. Withdrawal had high loading on component 3, suggesting that this represents withdrawal.

**TABLE 2:** Principal components of the Bush-Francis Catatonia Screening Instrument.

*n* = 1,456	Component 1 (parakinetic)	Component 2 (hypokinetic)	Component 3 (withdrawal)
**Component loadings**			
Excitement	0.24	−0.17	0.16
Immobility/stupor	−0.10	**0.36**	−0.24
Mutism	−0.12	**0.42**	0.36
Staring	0.18	0.24	−0.16
Posturing/catalepsy	**0.33**	0.18	−0.43
Grimacing	**0.38**	−0.03	0.00
Echopraxia/echolalia	**0.31**	−0.16	0.30
Stereotypy	**0.38**	−0.21	0.16
Mannerisms	**0.39**	−0.15	−0.01
Verbigeration	0.28	−0.07	0.08
Rigidity	0.26	**0.43**	−0.08
Negativism	0.21	**0.34**	0.33
Waxy flexibility	0.21	**0.39**	−0.02
Withdrawal	−0.12	0.17	**0.58**

*Coefficients with magnitude >0.3 are shaded and shown in bold. Where a clinical feature had a loading of >0.3 on more than one component, the coefficient for the component with the highest loading has been used.*

### Associations With Principal Components

The associations of principal components with gender, ethnicity, diagnostic group and treatment setting are shown in Table 3. In a linear regression of principal components on age in years, the coefficients were −0.01 (95% CI −0.015 to −0.006, *p* < 0.001) for the parakinetic component, 0.003 (−0.000 to 0.007, *p* = 0.07) for the hypokinetic component and −0.000 (−0.003 to 0.003, *p* = 0.97) for the withdrawal component. After interaction with peer reviewers, we explored associations between serum iron and creatine kinase, laboratory investigation results that we have previously found to be linked to catatonia (24), and the principal components (Supplementary Table 2). Creatine kinase was positively associated with the parakinetic component, but there were no other statistically significant associations.

**TABLE 3:** Principal component means by gender, ethnicity, diagnostic group, and treatment setting.

	Component 1 (parakinetic)		Component 2 (hypokinetic)		Component 3 (withdrawal)	
	Mean (95% CI)	*p*	Mean (95% CI)	*p*	Mean (95% CI)	*P*
**Gender^a^**		<0.001		0.19		<0.001
Men	**−0.18 (−0.28 to −0.09)**		0.04 (−0.05 to 0.14)		**0.16 (0.09 to 0.24)**	
Women	**0.15 (0.05 to 0.25)**		−0.04 (−0.12 to 0.05)		−0.13 (−0.21 to 0.05)	
**Ethnicity^b^**		0.94		0.08		0.22
White	−0.03 (−0.15 to 0.09)		−0.43 (−0.15 to 0.07)		0.03 (−0.07 to 0.12)	
Black	0.01 (−0.08 to 0.11)		0.01 (−0.07 to 0.96)		−0.00 (−0.08 to 0.08)	
Mixed	0.07 (−0.34 to 0.49)		**−0.41 (−0.66 to −0.15)**		−0.29 (−0.63 to 0.05)	
Asian	−0.06 (−0.40 to 0.29)		0.09 (−0.15 to 0.32)		0.15 (−0.07 to 0.36)	
Other	−0.07 (−0.35 to 0.20)		0.15 (−0.14 to 0.43)		−0.05 (−0.28 to 0.18)	
**Diagnostic group^b^**		<0.001		0.05		0.01
Organic disorder	−0.06 (−0.35 to 0.23)		0.31 (−0.09 to 0.70)		−0.12 (−0.43 to 0.18)	
Neurodevelopmental disorder	**0.59 (0.08 to 1.09)**		**−0.33 (−0.65 to −0.01)**		−0.17 (−0.50 to 0.17)	
Non-affective psychosis	0.08 (−0.02 to 0.18)		−0.02 (−0.11 to 0.07)		**−0.09 (−0.18 to −0.01)**	
Mood disorder	−0.29 (−0.44 to −0.14)		0.10 (−0.05 to 0.24)		**0.14 (0.00 to 0.27)**	
Neurotic disorder	−0.04 (−0.44 to 0.36)		0.12 (−0.21 to 0.44)		0.28 (−0.00 to 0.56)	
Personality disorder	−0.12 (−0.54 to 0.30)		−0.06 (−0.43 to 0.31)		−0.05 (−0.39 to 0.29)	
Substance use disorder	0.27 (−0.23 to 0.77)		−0.37 (−0.85 to 0.12)		−0.39 (−0.68 to −0.11)	
**Treatment setting^b^**		0.24		0.001		0.03
Community	−0.09 (−0.23 to 0.06)		**−0.19 (−0.31 to −0.06)**		**−0.16 (−0.29 to −0.04)**	
Home treatment team	0.16 (−0.33 to 0.65)		**−0.48 (−0.90 to −0.07)**		0.04 (−0.28 to 0.37)	
Psychiatric inpatient	0.00 (−0.09 to 0.10)		0.03 (−0.05 to 0.12)		0.06 (−0.28 to 0.14)	
Consultation-liaison psychiatry	−0.10 (−0.30 to 0.10)		0.09 (−0.10 to 0.28)		−0.03 (−0.20 to 0.13)	
Health-based place of safety	**0.52 (0.21 to 0.83)**		0.15 (−0.34 to 0.63)		−0.45 (−0.78 to 0.10)	

*Values in bold have 95% confidence intervals that do not include zero.*

*^a^p-values derived from unpaired t-test.*

*^b^p-values derived from ANOVA.*

In the cohort design, duration of admission and catatonia relapse were used as the dependent variables for linear regressions and logistic regressions, respectively, with the principal components as independent variables. Results are shown in Table 4.

**TABLE 4:** Linear regression of duration of admission on principal components and logistic regression of subsequent catatonia relapse on principal components.

	Duration of admission (days)^a^				Catatonia relapse			
	Unadjusted coefficient (95% CI)	*p*	Adjusted coefficient (95% CI)^b^	*p*	Unadjusted OR (95% CI)	*p*	Adjusted OR (95% CI)^b^	*p*
Component 1 (parakinetic)	**1.10 (1.02–1.19)**	**0.01**	**1.09 (1.01–1.17)**	**0.03**	1.03 (0.95**–**1.13)	0.48	1.03 (0.92**–**1.15)	0.59
Component 2 (hypokinetic)	1.06 (0.98**–**1.15)	0.16	1.08 (0.99**–**1.17)	0.09	**1.23 (1.12–1.36)**	<**0.001**	**1.18 (1.05–1.34)**	**0.01**
Component 3 (withdrawal)	**(0.84 (0.77–0.92)**	<**0.001**	**0.85 (0.78–0.93)**	<**0.001**	1.09 (0.97–1.21)	0.14	1.04 (0.91–1.19)	0.58

*Values in bold have 95% confidence intervals that do not include zero.*

*^a^Due to positive skew of duration of admission, a logarithmic transformation of admission duration was used. The displayed coefficients have been exponentiated.*

*^b^Adjusted for age, gender, ethnicity, and diagnostic group.*

### Phenomenology

In 68 patients, there was some account of the individual’s subjective experience, as shown in Table 5. Of these, 49 had some contemporaneous account and 24 provided a retrospective account, with five patients giving both. Among the patients with both contemporaneous and retrospective accounts, four out of five showed the same theme in both accounts. For example, motor passivity featured for one patient contemporaneously (“He describes a subjective sensation of being pushed in the opposite direction as if a force is repelling him.”) and retrospectively (“If you do not wait for the readiness, then it can feel like there is a bungee attached to you pulling you away from the door.”) However, four out of five patients elaborated on a theme in one account that was not present in the other. For example, a contemporaneous account was reported as “Could articulate that she was very scared but no detail on this,” while the retrospective account elicited the abnormal perception behind the fear: “She said she felt that there was another person behind her wanting to hurt her during the interview.”

**TABLE 5:** Categorisation of subjective experiences of catatonia.

				n	% (of 68)	Example text extract
**Timing of account**						
Contemporaneous				49	72	
	Awareness of catatonia			19	28	
Retrospective				24	35	
	Recollection of catatonia			21	31	
**Affective experience**						
Feeling unable to move				13	19	Feels “locked in.”
Subjective confusion				9	13	She reports feeling that her mind is clouded.
Distress				34	50	Standing in the same position for hours, voicing that it hurts, but will not allow himself to move.
	Fear			24	35	Feels “scared” and unsure what’s happening.
	Guilt			4	6	“What do I feel?.. I feel guilty… I’ve been on medications… I feel guilty… for what I’ve put you through.” She told me after several promptings that “I stole a baby.”
**Meaningful narrative**						
Any meaningful narrative explanation				49	72	His explanation [for writhing/flapping movements] is that he is “keeping his hands warm.”
Hallucinations				16	24	She reported that she could see a snake in the proximity near the receptionist, she reported that the snake was talking to her but she could not make out what it was saying to her.
	Auditory			13	19	He was mute earlier today because the voices were telling him his head would explode if he moved about.
		Commands		11	16	States that the voices are instructing to perform certain movements.
			Commands not to eat or drink	3	4	He made references to God wishing him to fast and didn’t eat or drink from that point onwards. […] He had been told by God he was Jesus Christ. Not speaking or eating was all on divine command.
	Visual			4	6	He went on to say that he had seen evil spirits/the devil. He sees these spirits enter people and believes they are bad people.
	Olfactory			2	3	She said there was also a smell as though something was burning, and she was trapped in the hall.
Delusional explanation				35	51	I met him kneeling on the floor with his forehead on the floor. He said he had adopted the position to save his life and kept asking to be seen by a neck doctor. […] He kept talking about his head falling off his neck.
	Grandiosity			3	4	Saying that she is the Golden Child who was meant to save the world, and she was not able to do so. […] Said that she could not move and was being instructed to stay in the same place but could not elaborate.
	Paranoia			15	22	Suffered from paranoid delusions and felt that her food had been poisoned by members of the staff. As a result she refused to eat.
	Parasitosis			1	1	“I had something inside me (a bacteria) that was moving around inside me.” After he had the experience of feeling a parasite enter his body, he knew that what he had to do was perform a series of rhythmic head rolling movements to get rid of the parasite. He says that when he was head-rolling he was in a bit of a trance.
	Nihilism (not including Cotard’s syndrome)			5	7	He also believes he has HIV and cancer and is going to die.
	Cotard’s syndrome			3	4	Last night thinking she was dead, asking for crash team.
	Passivity			11	16	At other times his other arm was held in a fixed postures instead. He described that this was because at times he does not have control over his body. […] The ether around his body influenced the position of his hand.
Non-psychotic explanation				7	10	Stating for example that she was on the floor in the corridor because she was feeling unwell and does not respond to staff because she is a “private person” and does not “just talk to people,” especially if they “come into her face and start saying ‘Hello, hello.”’

Among the retrospective accounts, 21/24 (88%) demonstrated some recollection of the catatonia, but among the contemporaneous accounts, only 19/49 (39%) clearly demonstrated awareness of the catatonia.

Around a third (24/68; 35%) of the patients expressed fear, but others noted other unpleasant emotions during the catatonia. The majority, however (49/68; 72%), did have some meaningful narrative explanation for the catatonia. These varied from hallucinations, such as commands not to eat or speak, to delusions, such as paranoia or passivity. One category added *a posteriori* was explanations for aspects of catatonia that were odd but not overtly psychotic, such as moving to keep oneself warm or not responding because of a wish to remain private.

There was no association between the presence of a meaningful narrative or subjective explanation for the catatonia with age (OR 1.01, 95% CI 0.97–1.05, *p* = 0.63), gender (OR 1.05, 95% CI 0.36–3.09, *p* = 0.92), ethnicity (*p* = 0.87) or diagnostic group (*p* = 0.23). There was no association between the presence of reported distress during catatonia with age (OR 0.98, 95% CI 0.95–1.02, *p* = 0.36), gender (OR 1.00, 95% CI 0.38–2.63, *p* = 1.00), ethnicity (*p* = 0.24) or diagnostic group (*p* = 0.94). However, the numbers were small.

## Discussion

### Summary of Findings

In one of the largest studies of catatonia psychopathology and phenomenology to date, we studied the catatonic signs recorded in case notes of 1,456 patients with catatonia, examining the phenomenology in a subgroup of 68 patients with available data. Cases were defined on the basis of a minimum of 2 reported catatonic signs and we found that the total number of reported signs had a median of 3 with a maximum of 14. The most commonly reported signs were mutism, immobility/stupor and withdrawal. Excitement was less common, but when it did occur, the same catatonic episode also featured immobility/stupor in approximately half of cases. Cluster analysis generated positive and negative groups of clinical features, while principal component analysis led to the identification of three components, which can broadly be considered as parakinetic features, hypokinetic features and withdrawal. The parakinetic component was associated with women, younger age, neurodevelopmental disorders and longer admission duration; the hypokinetic component was associated with catatonia relapse; the withdrawal component was associated with men and mood disorders.

In terms of the phenomenology, there was a very varied subjective experience of catatonia. Less than half of the patients expressed fear, though a few expressed other forms of distress. Others had potentially meaningful explanations for the catatonia, which ranged from hallucinations to delusions to seemingly non-psychotic rationalisations. Affective and meaningful narrative explanations for catatonia were not mutually exclusive.

### Limitations

This study’s main strengths are its large size, its breadth across diagnoses and treatment settings, and the independent validation of diagnoses of catatonia.

However, it has several limitations, many of which are related to the quality of the routine medical records on which it relies. Given that the aims were descriptive, it is possible that some of our findings arose due to chance.

Selection bias is likely to have occurred because some more atypical catatonic presentations may not have been identified by the treating clinicians as catatonia, so these would not have been detected in our search; for example, excitement could have been misattributed to other illnesses, akathisia or drugs, which were not adequately encoded in the structured fields of the healthcare records. Moreover, given the predominance of psychotic disorders, it is likely that a large proportion of patients were taking antipsychotic medications, so extra-pyramidal side effects might have mimicked catatonic signs such as stereotypies and mannerisms. The phenomenology study is likely to be at especially high risk of selection bias because the number of patients with relevant data was such a small subset of the overall sample that factors such as lucid descriptions may have been preferentially included. In terms of measurement, it is worth noting that the phenomenological descriptions had—in many cases—been interpreted to some extent by the clinician, so it is possible that some nuance or meaning was lost (or inserted) in this process. Certainly, there is a risk that clinicians preferentially recorded interesting or novel phenomenological accounts, or conversely accounts that adhered to their own understanding of catatonia, especially given that evaluating the subjective experience is not a routine part of clinical evaluation. There was also a process of retrospectively scoring clinical notes by the investigators, which may have introduced a bias toward our hypotheses. Given that there were some discrepancies between the coders, there is likely to have been some measurement error at the point of coding the psychopathology; this is probably reduced in the phenomenological data because every entry was coded by two independent raters with arbitration where necessary.

As most of this study is descriptive, there is less scope for confounding and Table 4 showed that the measured demographic and diagnostic confounders did not have a major impact, but it is possible that other confounders such as medical comorbidities or medications may have altered the associations.

In terms of the principal component analysis, it should be noted that the 3-component model that we used only explained 31.1% of the variance, so there is a substantial amount of information that is lost if this model is used. It is hard to draw any conclusions from associations with laboratory investigation results, as the number of patients with a valid result was very small.

One final limitation specific to our finding of psychomotor alternation is that we assessed the phenomenon of alternation with the two clinical features that are most intuitively identified with hypoactive and hyperactive states, stupor, and excitement. The concept of psychomotor alternation, as articulated by Shorter and Fink, while emphasising these clinical features, is somewhat broader and incorporates hypoactive features such as mutism and staring as well as hyperactive features such as impulsivity and verbigeration. It is possible, therefore, that we underestimate its frequency (16).

### Psychopathology

Stupor and excitement are seemingly opposite states, such that they have been used to define distinct subtypes of catatonia (33, 34). However, our study found that where excitement was present, stupor commonly occurred within the same catatonic episode. Our cluster analysis similarly suggests that essentially all patients who are identified as having catatonia have some of the negative features of the condition. Only 40.5% had a positive feature, however. These two analyses support the recent concept of psychomotor “alternation” in catatonia (16) and findings in organic catatonia of coexisting excitement and stuporous features (35). The whole first episode of catatonia was reviewed to identify catatonic features, which could have contributed to the relatively high rate of co-occurrence.

Unlike previous principal component analyses, all the patients included in our study met a diagnostic threshold for catatonia. Nonetheless, the finding of three components is consistent with other studies (4–7) and the nature of these components is similar. Our finding of a higher scoring on the withdrawal component in mood disorders is consistent with, though not quite the same as, a previous finding of higher “inhibition” (predominantly motor) in depression (8), although this study did not have a withdrawal item. Taken with Stuivenga and Morrens’ finding of higher excitement in psychotic disorders than in depression (10), our result of greater withdrawal in mood disorders does suggest that catatonia in depression is a more inhibited state than catatonia in primary psychotic disorders. The most extreme component scores were associated with diagnoses of neurodevelopmental disorders (more parakinetic features and fewer hypokinetic features), which have often been excluded from other studies of catatonia. Knowledge of these associations may prove useful diagnostically. Our finding of an association of parakinetic features with a longer inpatient admission and hypokinetic features and catatonia relapse could aid in treatment planning.

The reasons for these associations with specific components, however, are currently unclear. It is possible that the different phenotype of catatonia in neurodevelopmental disorders relates to a differential response to antipsychotic treatment, since—for example—individuals with learning disability are at higher risk of various extrapyramidal side effects (36). However, it is also possible that the propensity for hyperkinetic features is intrinsic to neurodevelopmental disorders, such as autism (37). It is possible that the associations with inpatient admission and relapse reflect the underlying diagnoses.

### Phenomenology

Just as this study finds a range of psychopathology within an individual, so there is a variability in the phenomenology. In their book, *The Madness of Fear*, Shorter and Fink used a historical analysis to show that catatonia has frequently been associated with, and may be driven by, fear (16). Heightened emotional states and in particular, fear, have long been proposed as a key part of catatonia, but few studies have systematically assessed them. We found that half of the patients with phenomenological descriptions reported experiencing significant distress at the time of their catatonia, with over a third of patients explicitly describing fear. These figures may be an underestimate, as we elected to code only those responses that explicitly described fear or distress and did not infer emotions from potentially distressing experiences such as persecutory delusions. Moskowitz has hypothesised that catatonia is a primitive prey response to fear or extreme stress, analogous to the tonic immobility defence strategy in certain animals (21). The text extract in Table 5 that describes a woman experiencing a visual hallucination of a snake during her catatonia fits particularly well with this theory. Others have suggested that catatonia is a somatic alternative to expressing extreme fear with language (19).

However, our study adds weight to the idea that catatonia is not solely a function of fear (4) and may relate to psychotic explanations (18). Cohen proposed three separate phenomenological groups in paediatric catatonia: the hyperanxious who are “frightened stiff”; those with adherence to delusional ideas, such as a belief that a repetitive action was necessary; and those with resistance to delusional thinking, such as when an individual resists an impulse by holding still (22). We found examples of all of these states in our phenomenological analysis. Our distinctive contribution in this area has been to show that the inward experience of catatonia is highly varied and is not restricted to command hallucinations or paranoid delusions. Hallucinations were noted to occur across three sensory modalities and delusions could reflect grandiosity, paranoia, parasitosis, nihilism, or passivity. Many of these potential affective and meaningful narrative explanations for catatonia co-existed.

Previous work has found an association between an experience of intense anxiety and response to lorazepam in catatonia (20, 38, 39). We did not find any statistically significant associations between phenomenological data and demographic or clinical variables, but the numbers were comparatively small.

### Implications

In terms of future research, there is a need to assess psychopathology and phenomenology prospectively alongside each other. These may then be correlated with treatment response. One such study is currently in progress in South Africa (40). Secondly, while there have been a number of functional neuroimaging and neurophysiological studies in catatonia, these studies tend to lack analyses based on dimensions of psychopathology or phenomenology, which would be important to ascertain more specific mechanisms. For example, is the relative paucity of subjective accounts of catatonia an important clue to distinguishing subtypes? Alternatively, does genuine lack of awareness point to an alteration of consciousness and perhaps subcortical pathophysiology, while the presence of any phenomenology might suggest a more cortical localisation? Thirdly, although psychological interventions are very challenging in acute catatonia, our findings of distinct affective and other psychopathological experiences in catatonia raise the prospect of psychological interventions in the prevention of—particularly relapsing—catatonia.

Regarding clinical applications, our study suggests that detailed analysis of psychopathology can be of value in diagnosing not only catatonia but also the condition underlying it, as parakinetic features suggest neurodevelopmental disorders while withdrawal features suggest mood disorders. Far from being a blank mental state, our study finds a rich phenomenology in catatonia, which should remind clinicians to treat patients with dignity and respect, even—maybe especially—those who have limited responsiveness. Our finding of a wide range of psychotic experiences in catatonia should prompt clinicians to spend time trying to elicit such features, including taking a collateral history wherever possible.

## Data Availability Statement

The datasets presented in this article are not readily available because data are owned by a third party, Maudsley Biomedical Research Centre (BRC), Clinical Records Interactive Search (CRIS) tool, which provides access to anonymised data derived from SLaM electronic medical records. These data can only be accessed by permitted individuals from within a secure firewall (i.e., the data cannot be sent elsewhere), in the same manner as the authors. Requests to access the datasets should be directed to cris.administrator@slam.nhs.uk.

## Ethics Statement

The studies involving human participants were reviewed and approved by the Oxfordshire C Research Ethics Committee. Written informed consent from the participants’ legal guardian/next of kin was not required to participate in this study in accordance with the national legislation and the institutional requirements.

## Author Contributions

JR and ED designed the project with support from AD, GL, and MZ. ED identified the phenomenological categories with support from JR. ED and LC-S put text extracts into phenomenological categories with support from JR. JR conducted the statistical analysis with support from GL and BC. ED wrote the initial draft of the manuscript, which was revised by JR. AA provided support in writing the manuscript. All authors reviewed and approved the final manuscript.

## Author Disclaimer

The views expressed are those of the authors and not necessarily those of the NHS, the NIHR or the Department of Health and Social Care.

## Conflict of Interest

MZ declares honoraria for a lecture from Eisai Co., Ltd. The remaining authors declare that the research was conducted in the absence of any commercial or financial relationships that could be construed as a potential conflict of interest.

## Publisher’s Note

All claims expressed in this article are solely those of the authors and do not necessarily represent those of their affiliated organizations, or those of the publisher, the editors and the reviewers. Any product that may be evaluated in this article, or claim that may be made by its manufacturer, is not guaranteed or endorsed by the publisher.
