# Supplementary material for: Catatonia Psychopathology and Phenomenology in a Large Dataset
Source: Front Psychiatry. 2022 May 23;13:886662. doi: 10.3389/fpsyt.2022.886662 (PMC9168075; doi:10.3389/fpsyt.2022.886662)
Supplement: Supplementary file 1 [file Table_1.DOCX]

Supplementary Material

# Supplementary table 1: 5-component principal component analysis scores

| *n* = 1,456 | Component 1 | Component 2 | Component 3 | Component 4 | Component 5 |
| --- | --- | --- | --- | --- | --- |
| Component loadings |  |  |  |  |  |
| - Excitement | 0.2396 | -0.1744 | 0.1646 | -0.5192 | 0.0606 |
| - Immobility/stupor | -0.101 | 0.3587 | -0.2381 | 0.0814 | 0.2547 |
| - Mutism | -0.1225 | 0.4169 | 0.3644 | 0.1929 | -0.0045 |
| - Staring | 0.175 | 0.2393 | -0.1598 | 0.3918 | 0.566 |
| - Posturing/catalepsy | 0.3262 | 0.184 | -0.4282 | 0.0122 | -0.1567 |
| - Grimacing | 0.379 | -0.0317 | 0.0027 | 0.2736 | 0.0468 |
| - Echopraxia/echolalia | 0.309 | -0.1631 | 0.3002 | 0.1813 | 0.1705 |
| - Stereotypy | 0.3765 | -0.2083 | 0.1617 | 0.2409 | -0.0329 |
| - Mannerisms | 0.3902 | -0.148 | -0.014 | 0.2464 | -0.3965 |
| - Verbigeration | 0.2816 | -0.0662 | 0.084 | -0.3987 | 0.5652 |
| - Rigidity | 0.2589 | 0.4347 | -0.076 | -0.1518 | -0.2096 |
| - Negativism | 0.2071 | 0.3368 | 0.3256 | -0.2085 | -0.0696 |
| - Waxy flexibility | 0.2113 | 0.3854 | -0.0213 | -0.2312 | -0.1661 |
| - Withdrawal | -0.1158 | 0.1667 | 0.582 | 0.1661 | -0.0347 |

# Supplementary table 2: Associations between principal components and laboratory investigation results

|  | *n* | Component 1 (parakinetic) | | Component 2 (hypokinetic) | | Component 3 (withdrawal) | |
| --- | --- | --- | --- | --- | --- | --- | --- |
|  |  | **Coefficient (95% CI)** | ***p*** | **Coefficient (95% CI)** | ***p*** | **Coefficient (95% CI)** | ***p*** |
| Iron (μmol/L) | 44 | 0.04 (-0.02 to 0.10) | 0.18 | 0.01 (-0.05 to 0.08) | 0.65 | -0.03 (-0.10 to 0.03) | 0.32 |
| Creatine kinase (IU/L) ^a^ | 61 | 1.35 (1.04 to 1.76) | 0.03 | 0.92 (0.72 to 1.17) | 0.49 | 0.94 (0.77 to 1.16) | 0.56 |

^a^ Due to positive skew of duration of creatine kinase, a logarithmic transformation of admission duration was used. The displayed coefficients have been exponentiated.
